# Integrative machine learning and single-cell analysis identifies nicotine-related diagnostic genes and myeloid remodeling in COPD

**DOI:** 10.3389/fimmu.2026.1880745

**Published:** 2026-07-27

**Authors:** Wei Zhang, Hongmei Sheng, Yueyue Shi, Yuhang Jiang, Haiying Peng

**Affiliations:** 1 Department of Respiratory and Critical Care Medicine, Chest Hospital, Tianjin University, Tianjin, China; 2 Department of Blood Transfusion, The Affiliated Cancer Hospital of Zhengzhou University and Henan Cancer Hospital, Zhengzhou, China; 3 Tianjin Chest Hospital, Tianjin University, Tianjin, China

**Keywords:** COPD, immune infiltration, machine learning, myeloid cells, nicotine, single-cell RNA sequencing

## Abstract

**Background:**

Cigarette smoke is a major risk factor for chronic obstructive pulmonary disease (COPD), but its toxicity results from multiple harmful constituents. Nicotine is a chemically defined and biologically active tobacco-related compound, yet its COPD-related molecular features remain unclear. This study aimed to identify nicotine-related transcriptomic signatures in COPD and explore their immune and single-cell relevance.

**Methods:**

Nicotine-related targets were collected from PubChem, ChEMBL, and SwissTargetPrediction. COPD transcriptomic data from GSE57148 were used to identify differentially expressed genes, which were intersected with nicotine-related targets to obtain candidate genes. CIBERSORT was applied to evaluate immune infiltration. LASSO regression and multiple machine learning algorithms were used to construct diagnostic models. Molecular subtypes were defined using consensus clustering based on SVM-derived important genes, followed by GSVA and GSEA. Single-cell RNA-seq data from GSE173896 were analyzed to characterize cell composition, gene set activity, cell-cell communication, and myeloid-cell trajectories. Molecular docking, scTenifoldKnk-based virtual knockout, and RT-qPCR validation were further performed.

**Results:**

A total of 132 nicotine-related target genes were obtained. Nicotine-related differentially expressed genes showed distinct expression patterns between COPD and control samples and were associated with immune cell infiltration. Machine learning analysis identified an SVM model with the best diagnostic performance, achieving an AUC of 0.923. Five key genes, including CHKA, EBP, RPS6KA5, NCSTN, and CHRM3, were ranked as the most important features. COPD samples were further divided into two molecular subtypes with different immune infiltration and pathway activity. Single-cell analysis showed that nicotine-related diagnostic gene activity was enriched mainly in epithelial, stromal, endothelial, and myeloid-lineage cells. Myeloid-cell reclustering revealed inflammatory, antigen-presenting, and lipid-metabolic states. Pseudotime analysis linked SVM-prioritized genes to myeloid-cell state transitions. Molecular docking suggested potential interactions between nicotine and key target proteins. RT-qPCR confirmed decreased expression of CHKA and EBP, and increased expression of CHRM3, NCSTN, and RPS6KA5 in COPD samples.

**Conclusion:**

This study identified a nicotine-related diagnostic gene panel associated with immune infiltration, molecular subtypes, and myeloid-cell remodeling in COPD. These findings provide insight into nicotine-related molecular alterations, while indicating that nicotine-related signals represent only one component of cigarette smoke-associated COPD biology.

## 1 Introduction

Chronic obstructive pulmonary disease (COPD) is a long-term inflammatory airway disease characterized by persistent respiratory symptoms, progressive airflow limitation, and repeated tissue injury (1, 2). Although spirometry remains essential for diagnosis (3), COPD is biologically heterogeneous. Patients with similar clinical severity may differ in inflammatory patterns, immune-cell composition, airway remodeling, and disease progression (2, 4). This heterogeneity limits the ability of conventional clinical indicators to fully reflect disease activity and creates a need for molecular markers that can capture the underlying biological diversity of COPD (4, 5).

Cigarette smoke exposure is one of the most important environmental risk factors for COPD (6). However, cigarette smoke is a complex mixture containing multiple harmful constituents, including particulate matter, aldehydes, polycyclic aromatic hydrocarbons, tobacco-specific nitrosamines, and oxidants. Therefore, nicotine should not be considered equivalent to the overall toxicity of cigarette smoke. In the present study, nicotine was selected as a focused molecular entry point rather than as a surrogate for total tobacco smoke exposure. This choice was based on its clearly defined chemical structure, available target information, and broad biological activity. Nicotine can interact with cholinergic and other receptor-mediated signaling systems and may influence airway epithelial responses, immune-cell activity, airway smooth muscle behavior, and tissue remodeling (7, 8). Experimental studies have further shown that nicotine-related receptor signaling can affect airway smooth muscle metabolism, endoplasmic reticulum stress, and proliferative remodeling, supporting its potential role in airway structural changes (9, 10). Thus, mapping nicotine-related targets in COPD may help identify a specific subset of tobacco-related molecular alterations, while not representing the full pathogenic effects of cigarette smoke.

Transcriptomic data provide an opportunity to identify nicotine-related genes that are altered in COPD, but single-layer differential expression analysis is not sufficient to explain disease complexity (11, 12). COPD involves immune imbalance, myeloid-cell activation, stromal remodeling, and altered cell-cell communication (13, 14). Therefore, nicotine-related genes need to be examined not only at the bulk transcriptomic level but also in relation to immune infiltration and single-cell cellular context. In addition, machine learning offers a practical way to prioritize diagnostic features from high-dimensional gene expression data (14–16). When combined with single-cell analysis, these models may help connect patient-level diagnostic signals with specific cellular compartments and dynamic cell states.

In this study, we integrated chemical target prediction, bulk transcriptomics, machine learning, molecular subtyping, and single-cell RNA-seq analysis to investigate nicotine-related molecular features in COPD. Nicotine-related targets were collected from PubChem, ChEMBL, and SwissTargetPrediction and were intersected with COPD-associated differentially expressed genes. LASSO regression and multiple machine learning algorithms were used to construct diagnostic models, and an SVM-based model was identified as the best-performing classifier. The key genes derived from this model were then used for molecular subtyping, immune infiltration analysis, and functional enrichment. Single-cell RNA-seq data were further analyzed to determine the cellular distribution of nicotine-related gene activity, with a focus on myeloid-lineage heterogeneity, cell-cell communication, and pseudotime trajectories. Finally, molecular docking, virtual gene knockout, and RT-qPCR validation were performed to support the biological relevance of the key genes. This integrative strategy provides a multi-level view of nicotine-related diagnostic genes and their potential roles in COPD immune remodeling.

## 2 Methods

### 2.1 Identification of nicotine-related chemical features and target gene set

The chemical structure and molecular information of nicotine were collected from the PubChem database (17). After searching for “Nicotine”, the corresponding canonical SMILES, structure files, and three-dimensional conformer data were downloaded. The obtained SMILES was further used as the input for target prediction. Nicotine-related targets were collected from two independent resources. Experimentally supported compound-target associations were retrieved from the ChEMBL database (18), whereas predicted targets were obtained using the SwissTargetPrediction platform (19). All target names were converted into official gene symbols, followed by removal of duplicated records. Finally, the gene sets derived from ChEMBL and SwissTargetPrediction were merged, and their union was defined as the final nicotine-related target gene set.

### 2.2 Screening of nicotine-related differentially expressed genes and immune infiltration analysis in COPD

The COPD-associated transcriptomic dataset GSE57148 was obtained from the GEO database and used as the bulk transcriptomic discovery cohort in this study. According to the sample phenotype information, the included samples were classified into COPD and normal control groups. After data normalization and preprocessing, differential expression analysis was conducted to identify genes that were significantly dysregulated in COPD within this discovery cohort. The resulting DEGs were then overlapped with the nicotine-related target gene set derived from ChEMBL and SwissTargetPrediction. Genes shared by both gene sets were considered nicotine-related DEGs in COPD and were retained as candidate genes for downstream analysis. To characterize the immune microenvironment associated with COPD, CIBERSORT was applied to estimate the relative proportions of immune cell subsets in each sample based on the normalized gene expression profiles. The distribution of immune cell infiltration was compared between COPD and normal samples. In addition, the relationship between nicotine-related DEGs and immune infiltration was evaluated by correlation analysis. Spearman correlation coefficients were calculated between candidate gene expression and the inferred abundance of immune cell subsets, thereby identifying potential links between nicotine-related molecular alterations and immune dysregulation in COPD.

### 2.3 Development of nicotine-related machine learning diagnostic models for COPD

LASSO logistic regression was first applied to reduce the dimensionality of nicotine-related candidate genes. The normalized gene expression matrix was used as input, and samples were labeled as COPD or control according to their phenotype information. Genes with zero variance were excluded, and missing values were replaced by the median expression value. LASSO regression was performed using the glmnet R package with a binomial model. Stratified cross-validation was used, and AUC was selected as the optimization metric. The lambda.1se criterion was used to determine the final penalty parameter, and genes with non-zero coefficients were retained for subsequent model construction. The LASSO-selected genes were then used to build diagnostic models using multiple machine learning algorithms, including logistic regression, random forest, support vector machine, XGBoost, gradient boosting machine, k-nearest neighbors, and naive Bayes. These algorithms were selected to cover different modeling assumptions and classifier types. Logistic regression was used as an interpretable linear baseline model; support vector machine was included as a kernel-based classifier suitable for high-dimensional data; random forest, XGBoost, and gradient boosting machine were used to capture nonlinear relationships and feature interactions through ensemble learning; k-nearest neighbors was included as an instance-based classifier; and naive Bayes was used as a simple probabilistic baseline model. The comparison of these algorithms was intended for exploratory model screening within the discovery cohort rather than for definitive clinical model validation. Model training was performed with the caret R package under 10-fold cross-validation. During preprocessing, near-zero variance features were removed, missing values were imputed, and expression values were centered and scaled. The diagnostic performance of each model was evaluated using AUC, accuracy, sensitivity, specificity, precision, and F1 score. The model with the highest AUC/C-index was selected as the best diagnostic model. ROC curves, predicted probability plots, model comparison bar plots, and variable importance plots were generated for model evaluation and interpretation.

In this study, the final SVM diagnostic model was constructed using the 19 genes selected by LASSO regression, rather than only the five top-ranked genes. Variable importance analysis was then performed within the SVM model to rank the contribution of these 19 features. CHKA, EBP, RPS6KA5, NCSTN, and CHRM3 were identified as the five highest-ranked explanatory genes and were selected for focused downstream analyses, including single-gene expression evaluation, pseudotime-related expression assessment, molecular docking, virtual knockout analysis, and RT-qPCR validation.

### 2.4 Molecular subtyping of COPD based on SVM-derived important genes

Consensus clustering was performed to identify molecular subtypes of COPD based on the important genes derived from the SVM model. The expression matrix of SVM-related genes was extracted from COPD samples and standardized using row-wise Z-score transformation. Extreme Z-score values were limited to the range of −2 to 2 to reduce the influence of outliers. Consensus clustering was conducted using the ConsensusClusterPlus (20) R package, with a maximum cluster number of 9, 50 resampling iterations, 80% sample resampling, k-means clustering, and Euclidean distance. Based on the clustering results, COPD samples were classified into two molecular subtypes, C1 and C2. PCA and heatmap analyses were used to visualize the separation and gene expression patterns of the identified subtypes.

### 2.5 Functional enrichment analysis of COPD molecular subtypes

Functional enrichment differences between C1 and C2 COPD subtypes were assessed using GSVA (21) and GSEA (22). For GSVA, KEGG and GO gene sets were used to calculate ssGSEA enrichment scores for each sample using the GSVA R package. Pathway activity differences between C2 and C1 were compared by t-test, and pathways with P < 0.05 were considered significant. For GSEA, genes were ranked according to the log2 fold change between C2 and C1, and enrichment analysis was performed using the clusterProfiler R package. Pathways with NES > 0 were considered enriched in C2, whereas pathways with NES < 0 were considered enriched in C1. Representative enriched pathways were visualized using bar plots and GSEA curves.

### 2.6 Single-cell RNA-seq data processing and cell-type annotation

Single-cell RNA-seq data of COPD were downloaded from the GEO database under accession number GSE173896. The raw expression matrix was processed using the Seurat (23–25) R package. Low-quality cells were removed according to quality-control metrics, including abnormal mitochondrial gene percentage, unreasonable numbers of detected genes, and abnormal total UMI counts. Potential doublets were also excluded before downstream analysis. After quality control, the remaining high-quality cells were normalized, and highly variable genes were identified. Batch effects across samples were corrected using the Harmony algorithm. Dimensionality reduction and clustering were performed based on the Harmony-corrected embeddings. Principal component analysis, neighbor graph construction, clustering, and UMAP visualization were conducted using the standard Seurat workflow. Cell clusters were annotated according to canonical marker genes of major cell types. The expression patterns of representative marker genes were visualized using the scCustomize package. Differentially expressed genes among cell populations were identified and further visualized using the scRNAtoolVis package. Functional enrichment analysis of marker genes from different cell types was performed and visualized using ClusterGVis (26), providing an overview of cell-type-specific biological programs in COPD.

### 2.7 Scoring of nicotine-related diagnostic genes in single-cell data

To evaluate the activity of nicotine-related diagnostic genes at the single-cell level, the LASSO-selected genes were used as a custom gene set. Five scoring methods were applied, including AUCell, UCell, singscore, ssGSEA, and Seurat AddModuleScore. AUCell scores were calculated based on gene expression rankings in each cell. UCell and singscore were computed using the irGSEA package (27), whereas ssGSEA scores were generated using the GSVA package. In addition, module scores were calculated using the built-in AddModuleScore function in Seurat. The scores obtained from the five methods were standardized and then normalized to a 0–1 range. An integrated gene set score was calculated by summing the normalized scores from all methods and was added to the metadata of the Seurat object. Dot plots and UMAP feature plots were used to visualize the distribution of gene set activity across cell populations.

### 2.8 Cell–cell communication and pseudotime trajectory analysis

Cell–cell communication analysis and pseudotime trajectory inference were performed to further characterize intercellular interactions and dynamic cellular states in COPD. For CellChat analysis, the annotated Seurat object was used as input, and cell-type information was extracted from the metadata. The CellChat (28) R package was applied to infer potential ligand–receptor interactions among different cell populations based on the CellChatDB database. Communication probability was calculated, and low-confidence interactions were filtered. Global interaction strength, cell-type-specific signaling networks, and major signaling pathways were visualized using built-in CellChat functions.

Pseudotime trajectory analysis was conducted using the Monocle2 R package. The expression matrix and cell annotation information were extracted from the Seurat object and converted into a CellDataSet object. Highly variable or differentially expressed genes were used for cell ordering. Dimensionality reduction was performed using the DDRTree algorithm, followed by cell trajectory construction and pseudotime estimation. Cells were visualized according to pseudotime, cell type, and cluster identity to explore potential state transitions among COPD-related cell populations.

### 2.9 Molecular docking analysis

Molecular docking was performed to evaluate the potential binding between nicotine and candidate target proteins. The three-dimensional structure of nicotine was obtained from PubChem, and protein structures of candidate targets were prepared as receptor files. Docking analysis was conducted using the online CB-Dock2 (29) platform, which supports protein–ligand blind docking by integrating cavity detection, docking, and homologous template fitting. The prepared receptor protein files and nicotine ligand file were uploaded to CB-Dock2 for docking prediction. Docking poses were ranked according to the predicted binding affinity, and the conformation with the lowest binding energy was selected as the optimal binding mode for each protein–ligand pair. The docking results were further used to assess the potential interaction between nicotine and key COPD-related target proteins.

### 2.10 *In silico* gene knockout analysis using scTenifoldKnk

In silico gene knockout analysis was performed using the scTenifoldKnk (30) R package to evaluate the potential regulatory impact of key genes in COPD-related cells. The raw count matrix of the selected cell population was extracted from the Seurat object and used as input. For each target gene, scTenifoldKnk was applied to simulate gene knockout at the single-cell transcriptomic level. Briefly, the algorithm constructed gene regulatory networks before and after virtual knockout, followed by manifold alignment to estimate transcriptomic perturbation induced by target gene deletion. Genes showing significant regulatory shifts after knockout were identified according to the perturbation score and adjusted statistical significance. The top dysregulated genes were retained for downstream functional interpretation, thereby helping to infer the potential regulatory role of key nicotine-related genes in COPD.

### 2.11 Virtual overexpression analysis

To evaluate the potential regulatory effects of key genes, virtual overexpression analysis was performed using scTenifoldOE. The raw counts matrix from single-cell RNA-seq data was used as input, and major cell types were randomly downsampled before analysis. Highly variable genes were selected and combined with each target gene to generate the input gene set. Virtual overexpression was separately performed for CHKA, EBP, RPS6KA5, NCSTN, and CHRM3, with the overexpression factor set to 10. scTenifoldOE constructed gene regulatory networks under wild-type and virtual overexpression conditions, followed by manifold alignment to estimate transcriptional perturbations induced by target gene overexpression. Genes with an adjusted P value < 0.05 were considered significantly affected genes. The top affected genes for each target were used for GO enrichment analysis.

### 2.12 RT-qPCR analysis

RT-qPCR was performed to validate the expression levels of candidate genes in clinical samples. A total of 20 clinical samples, including 10 control samples and 10 COPD samples, were included in the RT-qPCR validation cohort. The clinical characteristics of these samples, including age, sex, smoking status, and COPD diagnostic criteria, are summarized in **Supplementary Table 1**. Total RNA was extracted using TRIzol reagent, and RNA concentration and purity were assessed before reverse transcription. Complementary DNA was synthesized using a reverse transcription kit. Quantitative PCR was then performed on a real-time PCR system using SYBR Green-based reagents. GAPDH was used as the internal reference gene, and each sample was analyzed in triplicate. Relative gene expression levels were calculated using the 2^-ΔΔCt method. The primer sequences used in this study are listed in **Supplementary Table 2**. Differences between groups were evaluated using appropriate statistical methods, and P < 0.05 was considered statistically significant.

### 2.13 Statistical analysis

All statistical analyses were performed using R software. Differential expression analysis was conducted using the limma package, and multiple testing correction was performed using the Benjamini–Hochberg method when applicable. Correlations between gene expression and immune cell infiltration were assessed using Spearman correlation analysis. Machine learning models were evaluated by cross-validation, and diagnostic performance was assessed using AUC, accuracy, sensitivity, specificity, precision, and F1 score. For GSVA, pathway score differences between C1 and C2 were compared using Student’s t-test, while GSEA results were interpreted according to the normalized enrichment score. RT-qPCR data were analyzed using the 2^-ΔΔCt method, and group differences were assessed using Student’s t-test or Wilcoxon rank-sum test as appropriate. All statistical tests were two-sided, and P < 0.05 was considered statistically significant.

## 3 Results

### 3.1 Identification and functional annotation of nicotine-related target genes

**Figure 1:**
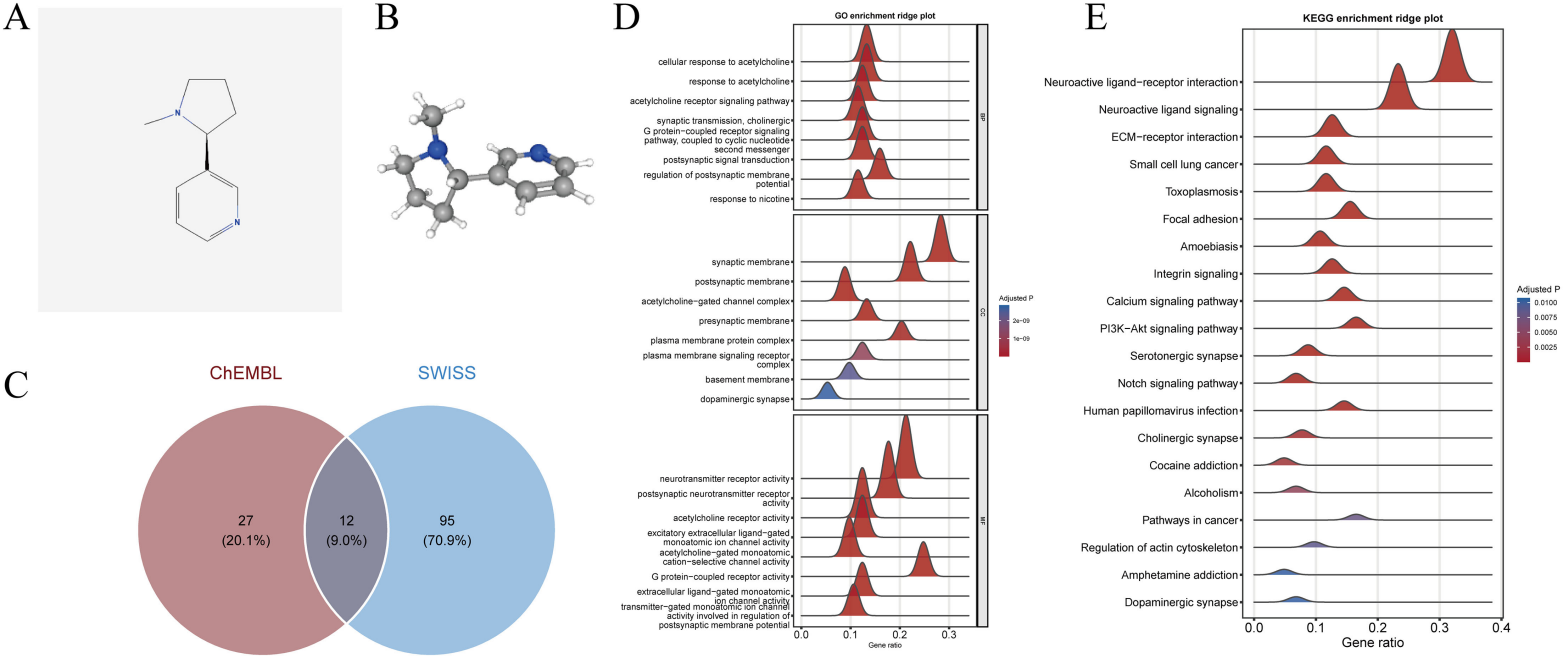
Chemical structure, target collection, and functional enrichment analysis of nicotine-related genes. **(A)** Two-dimensional chemical structure of nicotine obtained from PubChem. **(B)** Three-dimensional structure of nicotine. **(C)** Venn diagram showing the overlap of nicotine-related target genes obtained from ChEMBL and SwissTargetPrediction. **(D)** GO enrichment analysis of nicotine-related target genes. **(E)** KEGG enrichment analysis of nicotine-related target genes.

The chemical information of nicotine was first obtained from PubChem, including its two-dimensional and three-dimensional molecular structures (**Figures 1A, B**). To systematically collect nicotine-related targets, target genes were retrieved from both ChEMBL and SwissTargetPrediction. The Venn diagram showed that 27 genes were uniquely derived from ChEMBL, 93 genes were uniquely predicted by SwissTargetPrediction, and 12 genes were shared by the two sources. After merging these datasets and removing duplicates, 132 nicotine-related target genes were retained for subsequent analysis (**Figure 1C**). Considering the different evidence levels of the two sources, the targets derived from ChEMBL and SwissTargetPrediction were further documented separately. ChEMBL-derived targets represented experimentally supported or bioactivity-based associations, whereas SwissTargetPrediction-derived targets represented computationally predicted associations. The detailed gene lists from ChEMBL and SwissTargetPrediction are provided in **Supplementary Tables 3** and **4**, respectively. Functional enrichment analysis was then performed to explore the biological features of these nicotine-related targets. GO enrichment analysis showed that these genes were mainly associated with neurotransmitter receptor activity, synaptic membrane components, ligand-gated channel activity, acetylcholine receptor activity, and receptor-mediated signaling processes (**Figure 1D**). KEGG enrichment analysis further showed that these targets were involved in neuroactive ligand–receptor interaction, neurotrophin signaling pathway, ECM–receptor interaction, focal adhesion, calcium signaling pathway, PI3K–Akt signaling pathway, serotonergic synapse, cholinergic synapse, dopaminergic synapse, and addiction-related pathways (**Figure 1E**). These findings indicate that nicotine-related genes are closely connected with receptor signaling, neuronal regulation, cell adhesion, and intracellular signal transduction.

### 3.2 Nicotine-related differentially expressed genes were linked to immune remodeling in COPD

**Figure 2:**
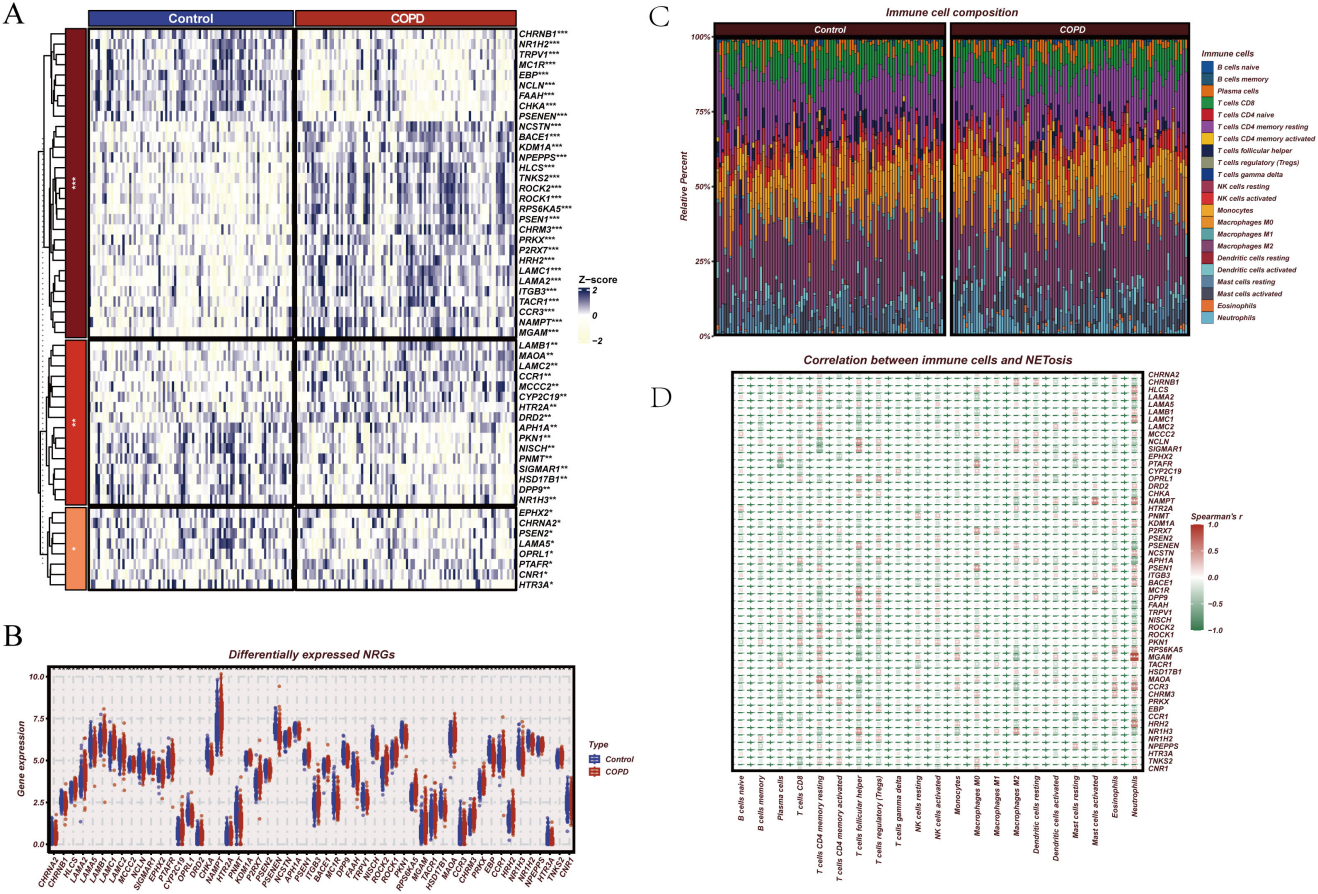
Differential expression of nicotine-related genes and immune infiltration analysis in COPD. **(A)** Heatmap showing the expression patterns of differentially expressed nicotine-related genes between control and COPD samples. **(B)** Boxplots showing the expression distribution of differentially expressed nicotine-related genes in the two groups. **(C)** Relative abundance of immune cell subsets estimated by CIBERSORT in each sample. **(D)** Correlation heatmap showing the associations between differentially expressed nicotine-related genes and immune cell infiltration levels.

After intersecting COPD-related DEGs identified from the GSE57148 discovery cohort with the nicotine-related target set, the retained genes showed a disease-associated expression pattern in this cohort. In the heatmap, control and COPD samples were separated into two groups according to sample annotation, and the nicotine-related DEGs formed three major expression blocks. The upper block showed stronger signals in COPD samples than in controls, suggesting that several nicotine-responsive genes were transcriptionally activated under the COPD condition. The middle block also displayed increased expression in the COPD group, although the signal was more heterogeneous across individual samples. By contrast, the lower block showed relatively higher expression in controls and weaker expression in COPD samples (**Figure 2A**). These patterns were consistent with the boxplot results, where many nicotine-related DEGs displayed visible shifts in median expression between the two groups rather than minor random fluctuations (**Figure 2B**). CIBERSORT analysis further showed that the immune composition differed considerably among samples. Macrophage subsets, CD4 T-cell populations, neutrophils, monocytes, and mast cell-related components contributed markedly to the inferred immune landscape. The COPD group did not show a simple replacement of one immune cell type by another. Instead, the stacked bar plot suggested a more complex immune redistribution, with noticeable sample-to-sample variation in both innate and adaptive immune components (**Figure 2C**). The correlation heatmap provided further evidence that nicotine-related DEGs were connected with immune infiltration. Most correlations were weak to moderate, but the overall pattern was not random. Several nicotine-related genes showed visible associations with macrophage subsets, neutrophils, monocytes, mast cells, and T-cell populations. The dense distribution of correlation signals across these immune compartments suggests that nicotine-related transcriptional changes may be involved in inflammatory cell recruitment, immune-cell activation, or immune imbalance in COPD (**Figure 2D**).

### 3.3 Machine learning identified an SVM-based diagnostic model with the best performance

**Figure 3:**
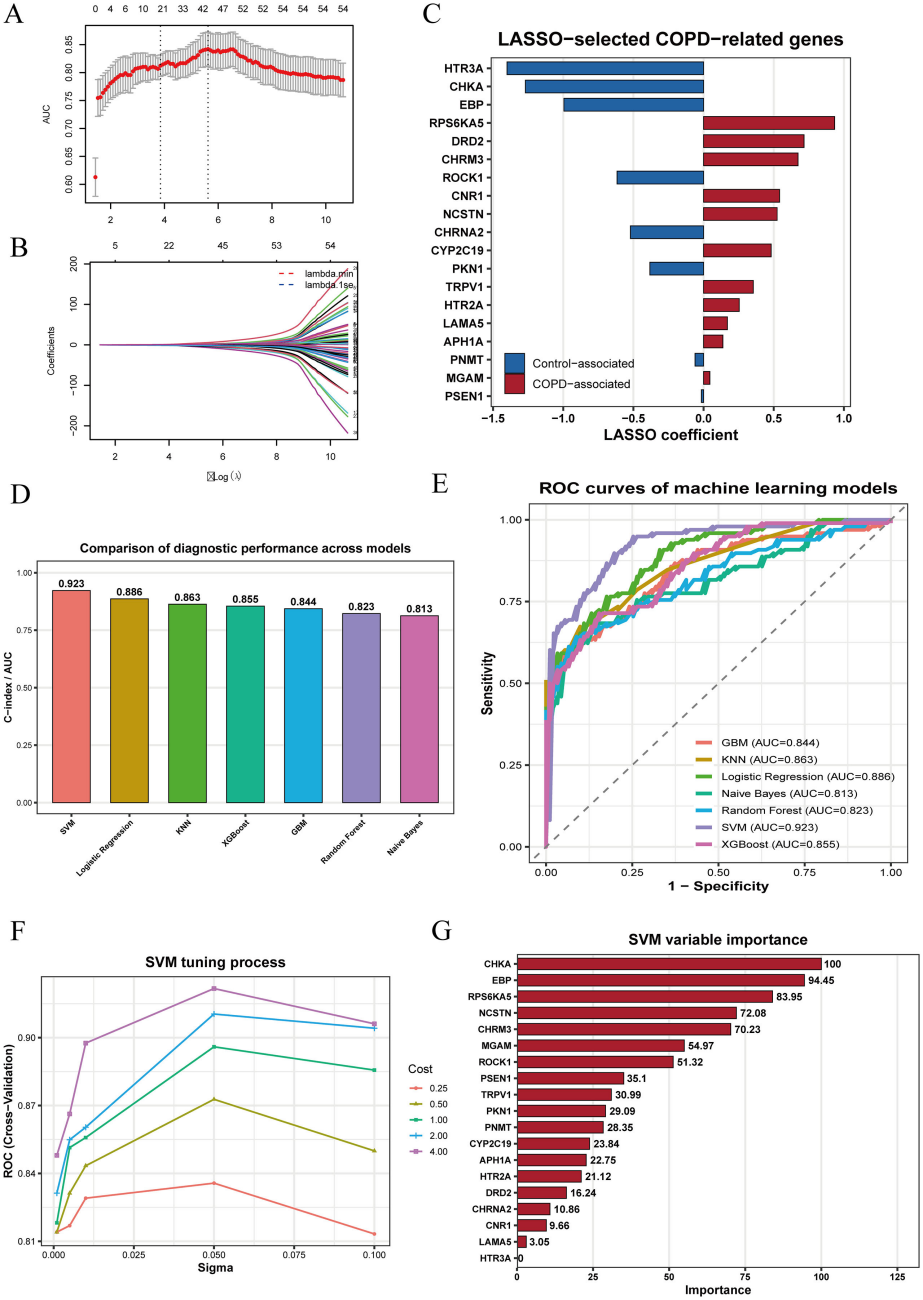
LASSO feature selection and machine learning construction of a COPD diagnostic model. **(A)** Cross-validation curve of the LASSO logistic regression model. **(B)** LASSO coefficient profiles of candidate genes. **(C)** Coefficients of LASSO-selected COPD-related genes, with red indicating COPD-associated genes and blue indicating control-associated genes. **(D)** Comparison of diagnostic performance across different machine learning models. **(E)** ROC curves of seven machine learning models. **(F)** Parameter tuning process of the SVM model. **(G)** Variable importance ranking of genes in the SVM model.

LASSO logistic regression was first used to reduce the number of nicotine-related candidate genes. In the cross-validation curve, the model performance increased as the penalty decreased and then reached a relatively stable range before showing mild fluctuation. The lambda.1se criterion was selected to obtain a more compact gene panel while maintaining acceptable diagnostic performance (**Figure 3A**). The coefficient path showed that candidate genes gradually entered the model as the regularization constraint was relaxed (**Figure 3B**). Using the lambda.1se cutoff, 19 genes were retained in the final LASSO model, including HTR3A, CHKA, EBP, RPS6KA5, DDR2, CHRM3, ROCK1, CNR1, NCSTN, CHRNA2, CYP2C19, PKN1, TRPV1, HTR2A, LAMA5, APH1A, PNMT, MGAM, and PSEN1. Among them, RPS6KA5, DDR2, CHRM3, CNR1, NCSTN, CYP2C19, TRPV1, HTR2A, LAMA5, PNMT, MGAM, and PSEN1 had positive coefficients, whereas HTR3A, CHKA, EBP, ROCK1, CHRNA2, PKN1, and APH1A showed negative coefficients (**Figure 3C**). The largest positive coefficients were observed for RPS6KA5, DDR2, and CHRM3, while HTR3A and CHKA showed the most prominent negative coefficients. The LASSO-selected genes were then used to train seven machine learning models. The SVM model achieved the highest diagnostic performance, with an AUC/C-index of 0.923. It should be noted that this diagnostic performance was obtained from the SVM model constructed using all 19 LASSO-selected genes. The five genes CHKA, EBP, RPS6KA5, NCSTN, and CHRM3 were not used alone to generate the reported AUC of 0.923. Instead, they were identified as the top-ranked explanatory features according to the variable importance analysis of the 19-gene SVM model. Logistic regression ranked second with an AUC of 0.886, followed by KNN (0.863), XGBoost (0.855), GBM (0.844), random forest (0.823), and naive Bayes (0.813) (**Figure 3D**). The ROC curves showed a similar pattern, with the SVM curve staying above the other models over a wide range of specificity thresholds (**Figure 3E**). For the SVM model, parameter tuning showed that ROC performance varied with both sigma and cost. The best performance was obtained at a relatively higher cost value and around sigma = 0.05, indicating that model tuning contributed to the final diagnostic accuracy (**Figure 3F**). Variable importance analysis further ranked the contribution of LASSO-selected genes in the SVM model. CHKA showed the highest importance score, followed by EBP, RPS6KA5, NCSTN, and CHRM3. These genes may represent the main contributors to the SVM-based COPD diagnostic classifier (**Figure 3G**). To further examine the expression relationships among the five SVM-prioritized genes, Spearman correlation analysis was performed across all included samples. CHKA was positively correlated with EBP, whereas both genes showed negative correlations with NCSTN, RPS6KA5, and CHRM3. In contrast, NCSTN, RPS6KA5, and CHRM3 displayed positive correlations with each other. These results suggest that the five core genes may represent two partially opposite expression patterns rather than a single redundant co-expression module (**Supplementary Figure 1**).

### 3.4 COPD samples were divided into two molecular subtypes with distinct gene expression and immune profiles

**Figure 4:**
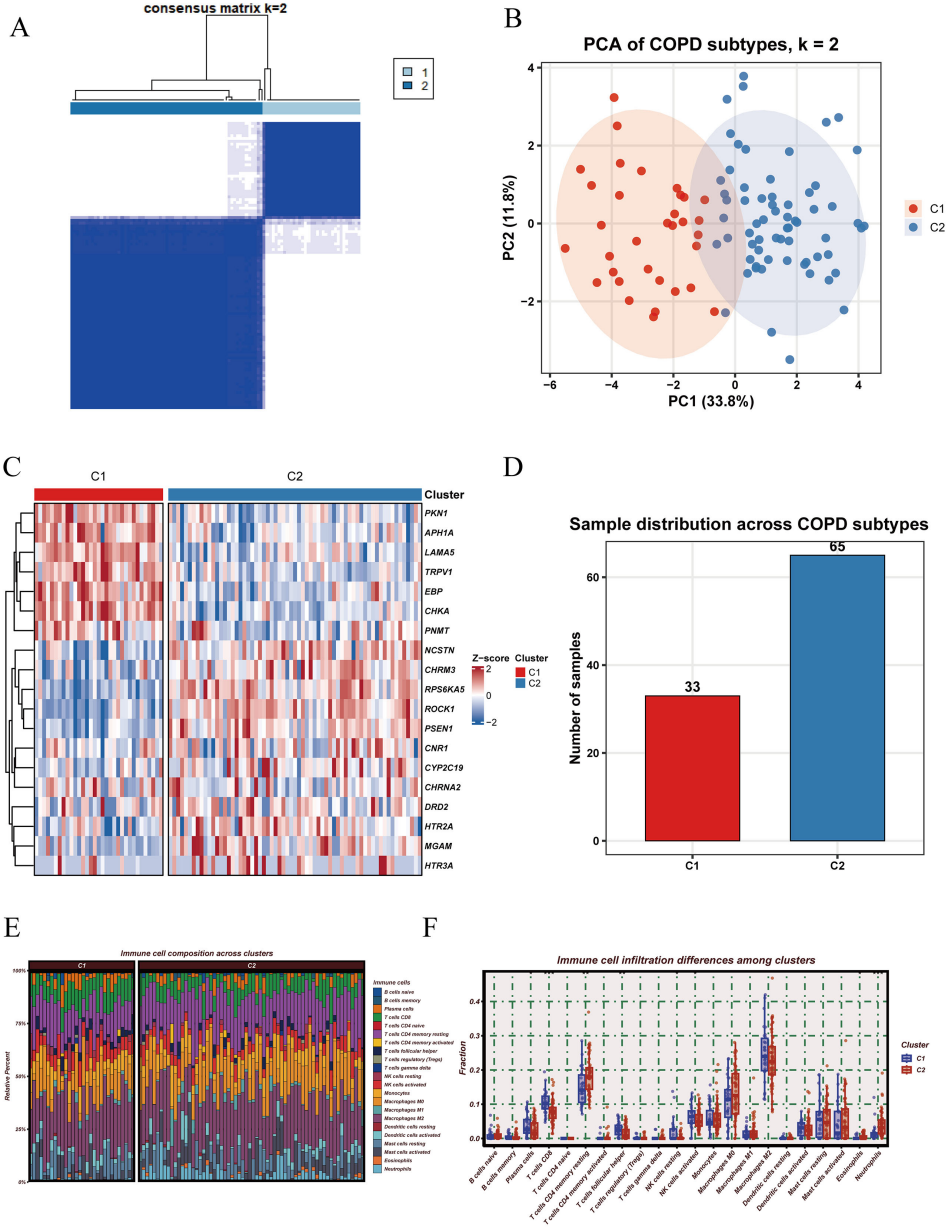
Consensus clustering and immune characterization of COPD molecular subtypes. **(A)** Consensus matrix showing the classification of COPD samples at k = 2. **(B)** PCA plot showing the distribution of C1 and C2 subtypes based on SVM-derived genes. **(C)** Heatmap showing the expression pattern of SVM-derived genes across the two subtypes. **(D)** Bar plot showing the number of samples in each subtype. **(E)** Stacked bar plot showing the immune cell composition of C1 and C2 samples estimated by CIBERSORT. **(F)** Violin plots comparing immune cell infiltration levels between C1 and C2 subtypes.

Consensus clustering was performed using SVM-derived important genes to further stratify COPD samples. When k was set to 2, the consensus matrix showed two relatively stable sample blocks, with high within-cluster consistency and limited overlap between clusters (**Figure 4A**). PCA also supported this classification. C1 samples were mainly distributed on the left side of PC1, whereas C2 samples were located more toward the right side. PC1 explained 33.8% of the variance, indicating that the subtype separation was mainly driven by the first principal component (**Figure 4B**). The two groups were not completely isolated, but the overall distribution showed a clear subtype tendency. The heatmap further showed that the two subtypes had different expression patterns of SVM-derived genes. PNMT, APH1A, LAMA5, TRPV1, EBP, CHKA, and PKN1 were more strongly expressed in C1, whereas NCSTN, CHRM3, RPS6KA5, ROCK1, PSEN1, CNR1, CYP2C19, CHRNA2, DDR2, HTR2A, MGAM, and HTR3A showed higher expression in C2 (**Figure 4C**). This expression structure was consistent with the PCA separation, suggesting that the SVM-derived genes retained subtype-discriminating information. Among all COPD samples, 33 were assigned to C1 and 65 were assigned to C2, showing that C2 represented the larger subtype in this cohort (**Figure 4D**). The immune infiltration analysis revealed visible differences in immune composition between the two subtypes. In the stacked bar plot, macrophage subsets, CD4 T-cell populations, neutrophils, monocytes, and mast cell-related components accounted for a large proportion of the inferred immune landscape (**Figure 4E**). The violin plots further showed that the two subtypes had different distributions of several immune cell subsets. C2 tended to show higher levels of monocytes, macrophage M0, and neutrophils, whereas C1 showed relatively higher distributions of CD8 T cells and macrophage M2 in several samples (**Figure 4F**). These results indicate that the molecular subtypes defined by SVM-derived genes were accompanied by different immune infiltration patterns.

### 3.5 Functional enrichment analysis revealed distinct biological programs between C1 and C2 subtypes

**Figure 5:**
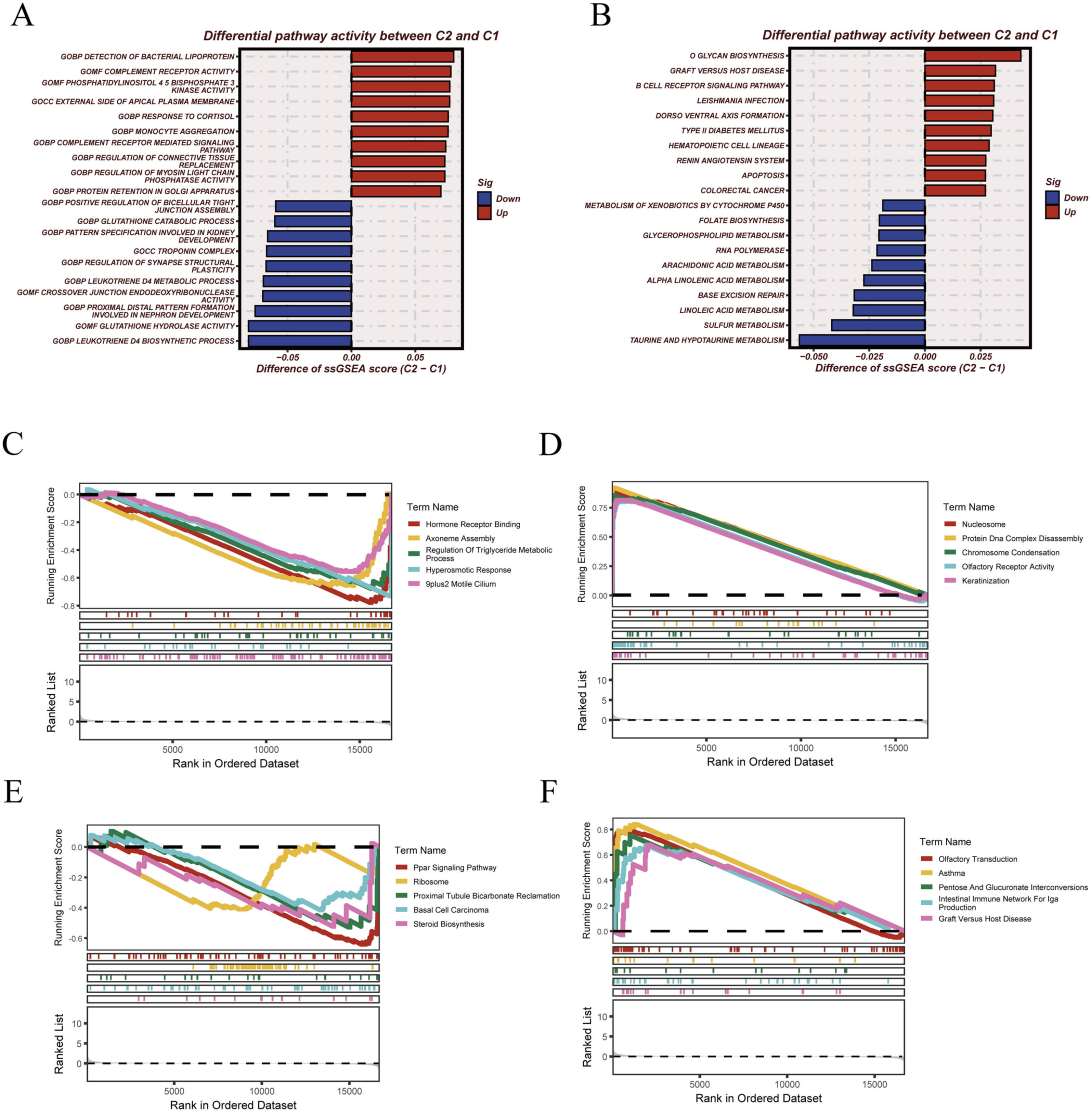
Functional enrichment analysis between C1 and C2 COPD molecular subtypes. **(A)** GO-based GSVA analysis showing differential pathway activity between C2 and C1. Red bars indicate pathways with higher activity in C2, and blue bars indicate pathways with higher activity in C1. **(B)** KEGG-based GSVA analysis showing differential pathway activity between C2 and C1. **(C, D)** GSEA plots showing representative GO terms enriched between C2 and C1. **(E, F)** GSEA plots showing representative KEGG pathways enriched between C2 and C1.

To clarify the functional differences between the two COPD molecular subtypes, GSVA and GSEA were performed based on GO and KEGG gene sets. GO-based GSVA showed that C2 had higher activity in immune- and receptor-related biological processes, including adaptive immune response, complement receptor activity, peptide-related immune regulation, and several muscle contraction or smooth muscle-associated terms. In contrast, C1 showed higher activity in pathways related to myeloid-cell migration, leukocyte activation, granulocyte chemotaxis, neutrophil chemotaxis, and regulation of inflammatory cytokine production (**Figure 5A**). This pattern suggests that C1 is more closely linked to inflammatory cell recruitment and innate immune activation, whereas C2 tends to show stronger adaptive immune and receptor-associated features.

KEGG-based GSVA further separated the two subtypes at the pathway level. C2 showed increased activity in immune-related pathways such as systemic lupus erythematosus, graft-versus-host disease, autoimmune thyroid disease, allograft rejection, hematopoietic cell lineage, and intestinal immune network for IgA production. By contrast, C1 displayed higher activity in metabolic pathways, including xenobiotic metabolism by cytochrome P450, drug metabolism, arachidonic acid metabolism, linoleic acid metabolism, alpha-linolenic acid metabolism, taurine and hypotaurine metabolism, and glutathione metabolism (**Figure 5B**).

GSEA provided additional support for the subtype-specific functional differences. GO enrichment curves showed that several biological processes related to receptor binding, hormone response, chromosome organization, and cellular structural regulation were unevenly distributed between C1 and C2 (**Figures 5C, D**). KEGG-GSEA results further highlighted differences in metabolic and immune-related pathways, including PPAR signaling, ribosome-related pathways, oxidative or metabolic regulation, olfactory transduction, and antigen processing-related processes (**Figures 5E, F**). Together, these results indicate that the two COPD molecular subtypes differ not only in diagnostic gene expression patterns but also in immune activation, inflammatory recruitment, and metabolic remodeling.

### 3.6 Single-cell analysis revealed COPD cellular composition and nicotine-related gene set activity

**Figure 6:**
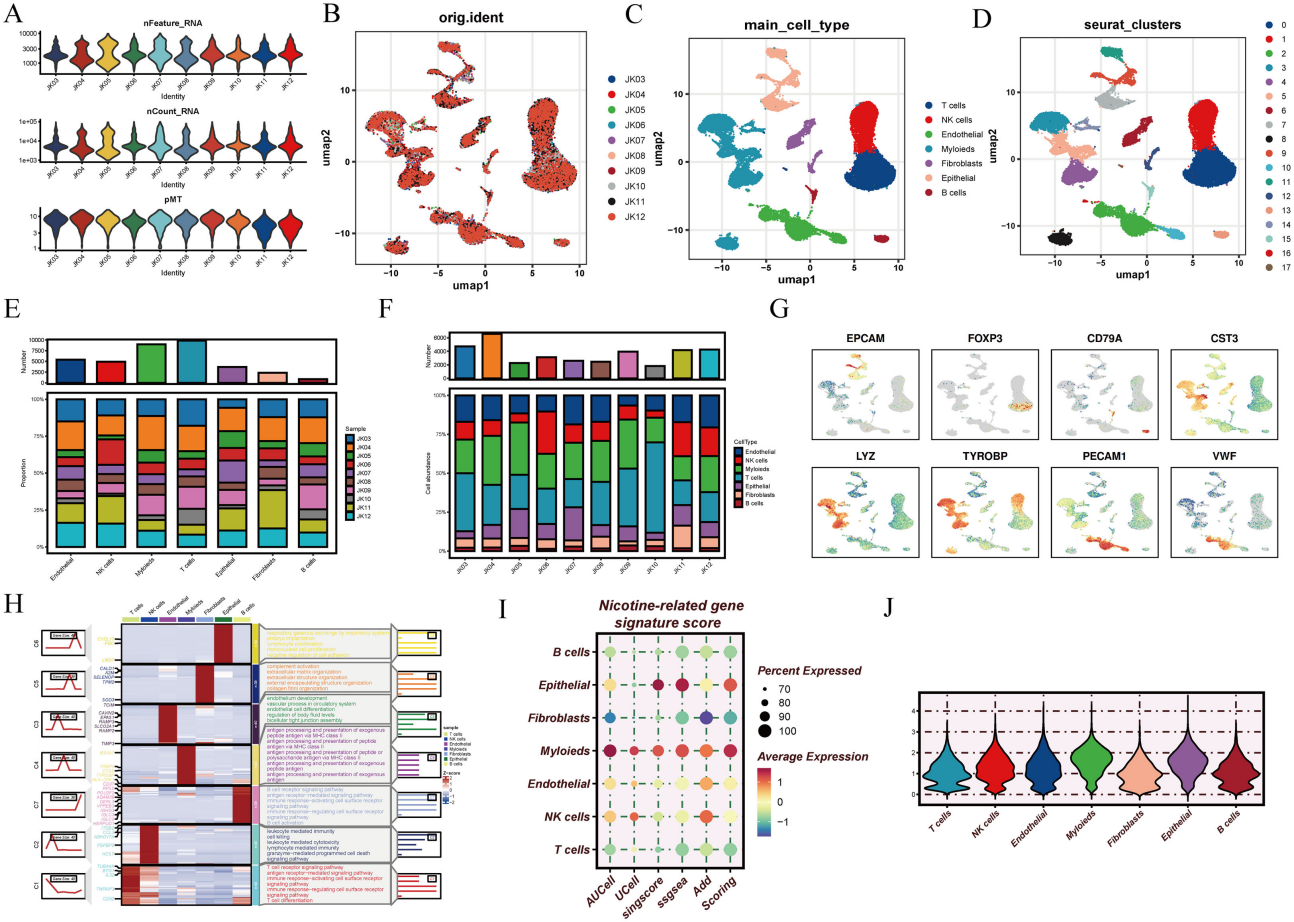
Single-cell transcriptomic landscape and nicotine-related gene set activity in COPD. **(A)** Violin plots showing quality-control metrics, including nFeature_RNA, nCount_RNA, and mitochondrial gene percentage across samples. **(B)** UMAP visualization of single cells colored by sample origin. **(C)** UMAP plot showing annotated major cell types. **(D)** UMAP plot showing Seurat clusters. **(E)** Cell-type composition across samples. **(F)** Relative proportion of major cell types in each sample. **(G)** Feature plots showing the expression of representative marker genes used for cell-type annotation. **(H)** Heatmap and functional annotation of cell-type-specific marker genes. **(I)** Dot plot showing nicotine-related gene set scores calculated by AUCell, UCell, singscore, ssGSEA, AddModuleScore, and the integrated scoring method across major cell types. **(J)** Violin plot showing the distribution of the integrated nicotine-related gene set score across cell types.

Single-cell RNA-seq data from GSE173896 were analyzed after quality control. The distributions of nFeature_RNA, nCount_RNA, and mitochondrial gene percentage were generally comparable across samples after filtering, although several samples still showed differences in detected gene numbers and UMI counts, reflecting sample-level heterogeneity in sequencing depth and cellular complexity (**Figure 6A**). After batch correction and dimensionality reduction, cells from different samples were projected into a shared UMAP space rather than being separated only by sample origin, suggesting that the main structure of the dataset was driven by biological cell identity rather than technical batch effects (**Figure 6B**).

Based on canonical marker genes, the cells were annotated into seven major cell populations, including T cells, NK cells, monocytes, macrophages, fibroblasts, endothelial cells, and B cells (**Figure 6C**). The Seurat clustering result showed a finer subdivision of these populations, with immune and stromal cell compartments occupying distinct UMAP regions (**Figure 6D**). The cell composition plots further showed that myeloid-lineage cells, including monocytes and macrophages, accounted for a large fraction of many samples, whereas lymphoid cells and stromal cells varied across individuals (**Figures 6E, F**).

The marker gene expression patterns supported the cell annotation. LYZ and TYROBP were enriched in myeloid populations, PECAM1 and VWF marked endothelial cells, CD79A was mainly detected in B cells, and EPCAM was restricted to epithelial-related cells. FOXP3 showed a more limited distribution within the T-cell compartment, while CST3 was broadly expressed in myeloid-associated regions (**Figure 6G**). Functional annotation of cell-type-specific marker genes showed that different cell populations were associated with distinct biological programs, including immune activation, inflammatory response, extracellular matrix organization, endothelial function, and lymphocyte-related processes (**Figure 6H**).

The nicotine-related diagnostic gene set was then scored at the single-cell level using five independent methods and an integrated scoring strategy. In the dot plot, the gene set activity was not evenly distributed across cell types. Epithelial cells, fibroblasts, myeloid cells, and endothelial cells showed relatively higher scoring signals, whereas T cells, NK cells, and B cells displayed weaker activity across most scoring methods (**Figure 6I**). Although epithelial-lineage cells showed detectable nicotine-related gene set activity, this population was relatively underrepresented in the GSE173896 dataset after quality control. Therefore, epithelial cells were not further subdivided into AT1, AT2, club, ciliated, or basal cell subtypes in this study. The epithelial-cell-related signal was interpreted as a preliminary observation, whereas subsequent analyses focused mainly on myeloid-lineage cells, which showed higher representation and clearer nicotine-related activity in this dataset. The integrated score showed a similar pattern in the violin plot, with higher score distributions in monocytes/macrophages and stromal-related populations, especially fibroblasts and endothelial cells (**Figure 6J**). These results suggest that nicotine-related diagnostic genes may be more closely associated with epithelial injury, myeloid inflammation, and stromal remodeling in COPD.

### 3.7 NRGs-high samples showed stronger and broader intercellular communication activity

CellChat analysis was performed to compare cell–cell communication patterns between the NRGs-low and NRGs-high groups. In the outgoing signaling heatmaps, the NRGs-low group showed a relatively scattered communication pattern, with several matrix- and adhesion-related signals distributed mainly in endothelial cells, fibroblasts, epithelial cells, and myeloid cells. By contrast, the NRGs-high group displayed a denser signaling landscape. Multiple pathways, including extracellular matrix-related signals such as COLLAGEN, LAMININ, and FN1, as well as immune- or receptor-related pathways such as MHC, APP, CD99, CCL, CXCL, and TNF-related signaling, were more broadly represented across cell types (**Supplementary Figure 2A**).The incoming signaling pattern showed a similar trend. Compared with the NRGs-low group, the NRGs-high group had more widespread signal-receiving activity, especially in myeloid cells, endothelial cells, epithelial cells, and fibroblasts. This suggests that the high-score group was characterized not only by stronger signal secretion but also by enhanced responsiveness to signals from other cellular compartments (**Supplementary Figure 2B**).

The global communication strength plots further supported this difference. In the NRGs-low group, fibroblasts showed the strongest outgoing interaction strength, whereas endothelial cells showed the highest incoming strength. Myeloid cells, T cells, NK cells, and epithelial cells were positioned in the middle range, while B cells showed the weakest communication activity (**Supplementary Figure 2C**). In the NRGs-high group, the overall interaction strength increased. Fibroblasts still showed the highest outgoing strength, while myeloid cells displayed the highest incoming strength. Endothelial and epithelial cells also shifted upward in incoming interaction strength compared with the low-score group (**Supplementary Figure 2D**). These findings indicate that increased NRGs activity is accompanied by a more active intercellular communication network, particularly involving fibroblasts, myeloid cells, endothelial cells, and epithelial cells.

### 3.8 Myeloid subclustering revealed distinct inflammatory and metabolic states in COPD

Because the nicotine-related diagnostic gene set showed higher activity in myeloid-lineage cells, this compartment was further extracted and reclustered to resolve myeloid heterogeneity and to identify the specific subpopulations associated with nicotine-related transcriptional activity in COPD.

**Figure 7:**
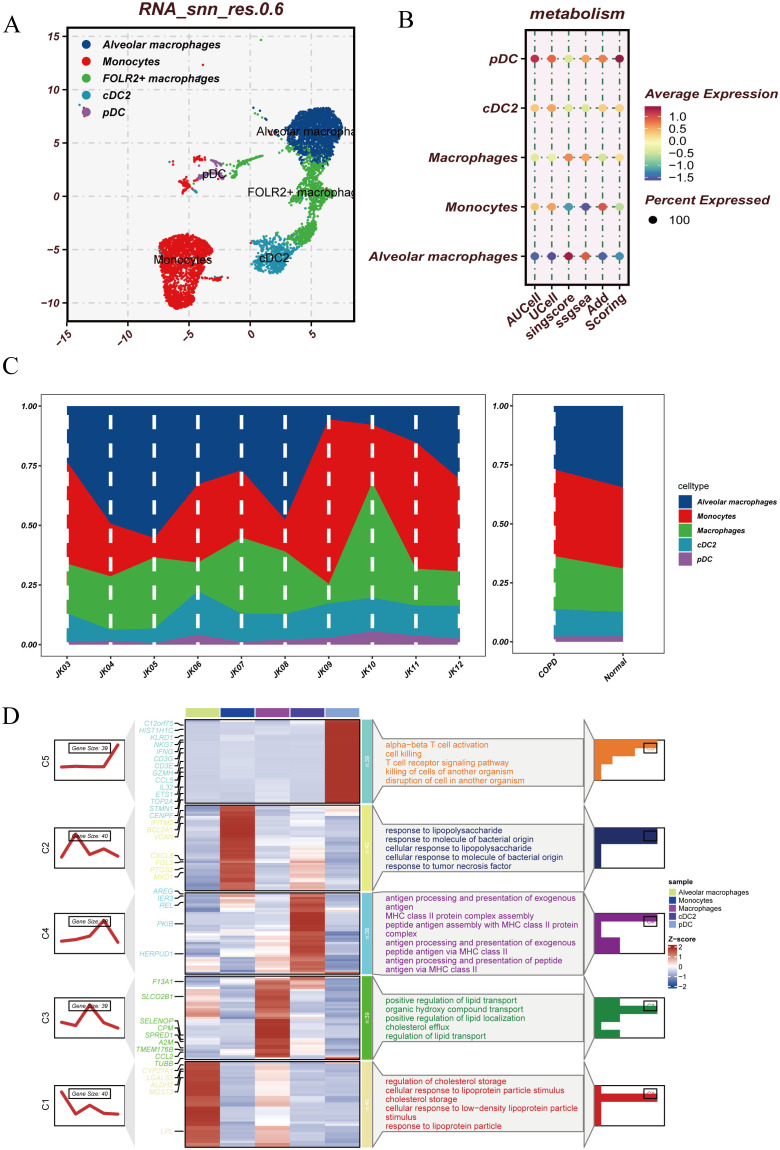
Subclustering and functional characterization of myeloid-lineage cells in COPD single-cell data. **(A)** UMAP visualization of myeloid-lineage cells after subclustering, including alveolar macrophages, monocytes, FOLR2+ macrophages, cDC2, and pDC. **(B)** Dot plot showing nicotine-related diagnostic gene set scores calculated by AUCell, UCell, singscore, ssGSEA, AddModuleScore, and the integrated scoring method across myeloid subtypes. **(C)** Stacked bar plots showing the relative proportions of myeloid subtypes across individual samples and disease groups. **(D)** Heatmap showing marker gene modules of myeloid subtypes and their corresponding functional enrichment terms.

Five major myeloid populations were identified, including alveolar macrophages, monocytes, FOLR2+ macrophages, cDC2, and pDC. On the UMAP plot, monocytes formed a compact cluster in the lower-left region, while alveolar macrophages were mainly located in the upper-right region. FOLR2+ macrophages occupied an intermediate area connected with other myeloid populations, and cDC2 cells formed a separate cluster adjacent to the macrophage compartment. pDC cells represented a small population and were distributed close to the central region of the myeloid map (**Figure 7A**). The annotation of these myeloid subpopulations was further supported by classical marker expression. Monocytes showed high expression of LYZ, S100A8, S100A9, and FCN1. Alveolar macrophages were characterized by MARCO, FABP4, PPARG, and MRC1. The FOLR2+ macrophage cluster showed higher expression of FOLR2, APOE, SELENOP, and other macrophage-associated genes, distinguishing it from the MARCO/FABP4/PPARG-positive alveolar macrophage population. cDC2 cells were marked by FCER1A, CD1C, and CLEC10A, while pDC cells showed expression of LILRA4, TCF4, and IRF7. These marker patterns supported the reliability of the myeloid subtype annotation used in the downstream analyses (**Supplementary Figure 3**). Nicotine-related diagnostic gene set scoring showed clear differences among these myeloid subtypes. pDC displayed the strongest scoring signal across the six scoring approaches, indicating relatively high nicotine-related diagnostic gene set activity in this small population. cDC2 and FOLR2+ macrophages showed moderate activity, whereas alveolar macrophages had lower scores in most methods. Monocytes showed method-dependent variation, but the integrated score still separated them from alveolar macrophages (**Figure 7B**). Cell proportion analysis showed marked sample-level variation in the myeloid compartment. Monocytes and alveolar macrophages accounted for a large fraction of the myeloid cells in most samples, but their proportions fluctuated substantially across individuals. One sample showed a pronounced expansion of FOLR2+ macrophages, whereas several samples were dominated by alveolar macrophages or monocytes. At the group level, COPD and normal samples had broadly similar myeloid compositions, with only modest shifts in monocyte, FOLR2+ macrophage, and alveolar macrophage fractions (**Figure 7C**).

The marker gene heatmap and enrichment analysis further supported functional specialization among myeloid subtypes. The pDC-enriched module was linked to T-cell activation and immune effector-related processes. Monocyte/FOLR2+ macrophage-associated modules were enriched in responses to lipopolysaccharide, bacterial molecules, and tumor necrosis factor. Another module was characterized by antigen processing and presentation, especially MHC class II-related terms. Lipid-related programs, including cholesterol storage, cholesterol efflux, lipid transport, and responses to lipoprotein particles, were mainly observed in FOLR2+ macrophage- and alveolar macrophage-related modules (**Figure 7D**). These findings indicate that COPD myeloid cells contain inflammatory, antigen-presenting, and lipid-metabolic states, rather than a single uniform macrophage phenotype.

### 3.9 Pseudotime trajectory analysis linked SVM-prioritized genes to a myeloid transcriptional continuum

**Figure 8:**
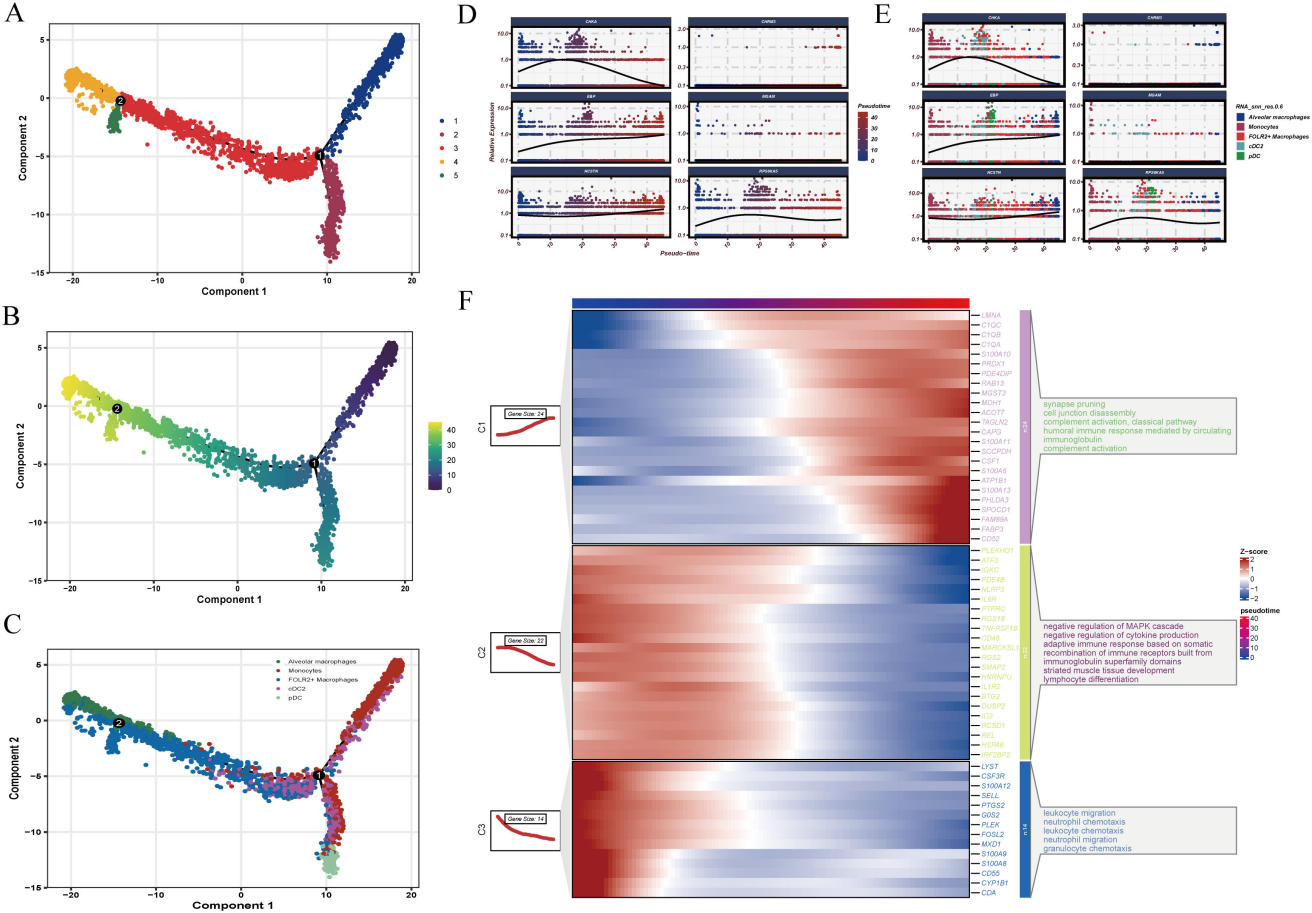
Inferred pseudotime trajectory analysis of myeloid-lineage cells in COPD. **(A)** Monocle2 trajectory plot showing myeloid cells colored by inferred cell state. **(B)** Pseudotime distribution of myeloid cells along the inferred trajectory. **(C)** Trajectory plot showing the distribution of myeloid subtypes, including alveolar macrophages, monocytes, FOLR2+ macrophages, cDC2, and pDC. **(D)** Pseudotime-associated expression patterns of the top five genes identified by the SVM model. **(E)** Expression patterns of the top five SVM-ranked genes across different myeloid subtypes along pseudotime. **(F)** Heatmap showing pseudotime-associated gene expression modules and their corresponding functional enrichment terms.

Because the nicotine-related diagnostic gene set showed relatively high activity in myeloid-lineage cells, this compartment was further extracted for pseudotime analysis. Monocle2 reconstructed a branched trajectory, suggesting a transcriptional continuum among myeloid-lineage cells. When colored by cell state, different inferred states occupied distinct regions of the trajectory, with state 1 mainly distributed along the upper-right branch, state 2 located along the lower-right branch, state 3 extending through the central trunk, and states 4–5 positioned near the left terminal region (**Figure 8A**). The pseudotime gradient further indicated ordered transcriptional variation across the trajectory, but this computational ordering should not be interpreted as direct evidence of an actual differentiation direction (**Figure 8B**).

Mapping annotated myeloid subtypes onto the trajectory showed that monocytes, FOLR2+ macrophages, alveolar macrophages, cDC2, and pDC occupied different but partially connected regions of the inferred continuum. Monocytes were mainly located in one branch of the trajectory, FOLR2+ macrophages were broadly distributed across the central region, and alveolar macrophages were enriched toward another branch. cDC2 and pDC showed more restricted distributions. These patterns suggest a state transition-like organization of myeloid cells in COPD, rather than proving a fixed lineage differentiation process (**Figure 8C**).

The expression dynamics of representative SVM-ranked genes were then examined along pseudotime. CHKA, EBP, NCSTN, RPS6KA5, CHRM3, and MGAM showed different pseudotime-associated expression patterns, indicating that these model-related genes were not uniformly distributed across the inferred myeloid continuum (**Figure 8D**). When colored by myeloid subtype, these genes also showed subtype-related distribution patterns along the trajectory. Signals of several genes were mainly observed in monocytes, FOLR2+ macrophages, and alveolar macrophages, whereas cDC2 and pDC contributed more restricted signals. This suggests that the SVM-ranked genes may be associated with specific myeloid states rather than reflecting a homogeneous myeloid-wide expression pattern (**Figure 8E**).

Pseudotime-dependent genes were then grouped into three major expression modules. The first module gradually increased along pseudotime and was enriched in cell migration, cell junction remodeling, extracellular structure organization, and wound response. The second module was more active at earlier or intermediate trajectory stages and was associated with immune regulation, including JAK–STAT signaling and inflammatory response-related processes. The third module showed a decreasing trend along pseudotime and was enriched in leukocyte migration, neutrophil chemotaxis, leukocyte chemotaxis, neutrophil migration, and granulocyte chemotaxis (**Figure 8F**). Together, these findings indicate that the inferred myeloid transcriptional continuum in COPD is accompanied by coordinated changes in inflammatory recruitment, immune regulation, tissue-remodeling programs, and subtype-associated expression of SVM-ranked genes.

### 3.10 Molecular docking and single-cell expression patterns supported the relevance of key model genes

**Figure 9:**
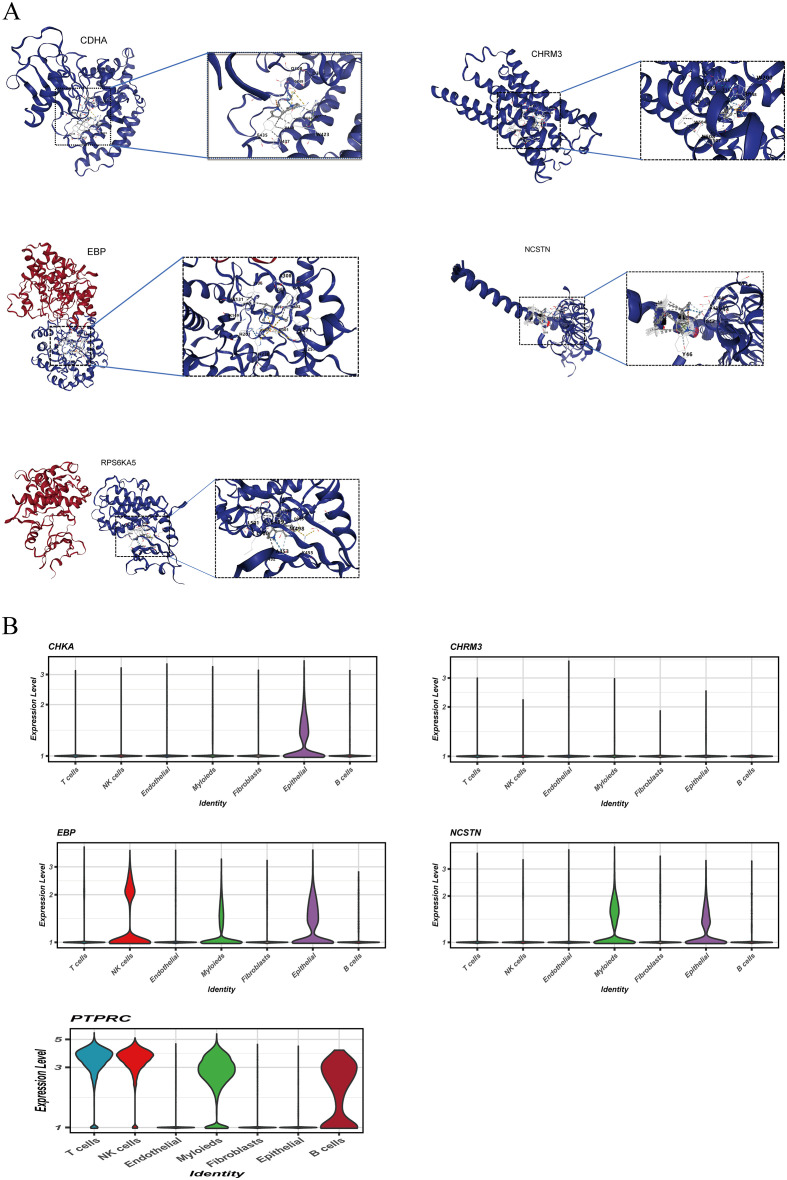
Molecular docking prediction and single-cell expression distribution of key diagnostic genes. **(A)** Molecular docking prediction of nicotine with proteins encoded by key SVM-prioritized genes, including CHKA, CHRM3, EBP, NCSTN, and RPS6KA5. The enlarged panels show the predicted binding pockets and local binding regions. **(B)** Violin plots showing the single-cell expression distribution of CHKA, CHRM3, EBP, NCSTN, and PTPRC across major cell types.

To further explore whether nicotine may have potential binding affinity with proteins encoded by the SVM-prioritized genes, molecular docking was performed as an exploratory structural analysis. The docking results showed that nicotine could be placed into the predicted binding pockets of CHKA, CHRM3, EBP, NCSTN, and RPS6KA5. In the enlarged local views, nicotine was located close to several amino acid residues within the predicted binding regions (**Figure 9A**). These results should be interpreted as hypothesis-generating structural evidence, rather than direct experimental confirmation of nicotine–protein interactions.

The single-cell expression distribution of representative genes was then examined across major cell populations. CHKA showed a relatively restricted expression pattern and was most evident in endothelial cells. CHRM3 was expressed at a low level across most cell types. EBP showed stronger expression in NK cells, macrophages, and endothelial cells, with a visible signal also detected in B cells. NCSTN was mainly detected in fibroblasts and endothelial cells. PTPRC, used as a pan-immune marker, was broadly expressed in immune cell populations, including T cells, NK cells, monocytes, macrophages, and B cells, but was nearly absent in fibroblasts and endothelial cells (**Figure 9B**). These results indicate that the key diagnostic genes were not uniformly expressed across all cells but showed cell-type-preferential expression patterns, linking the machine learning-derived genes to specific cellular compartments in COPD.

### 3.11 Experimental validation and in silico perturbation analysis supported the regulatory relevance of key genes

**Figure 10:**
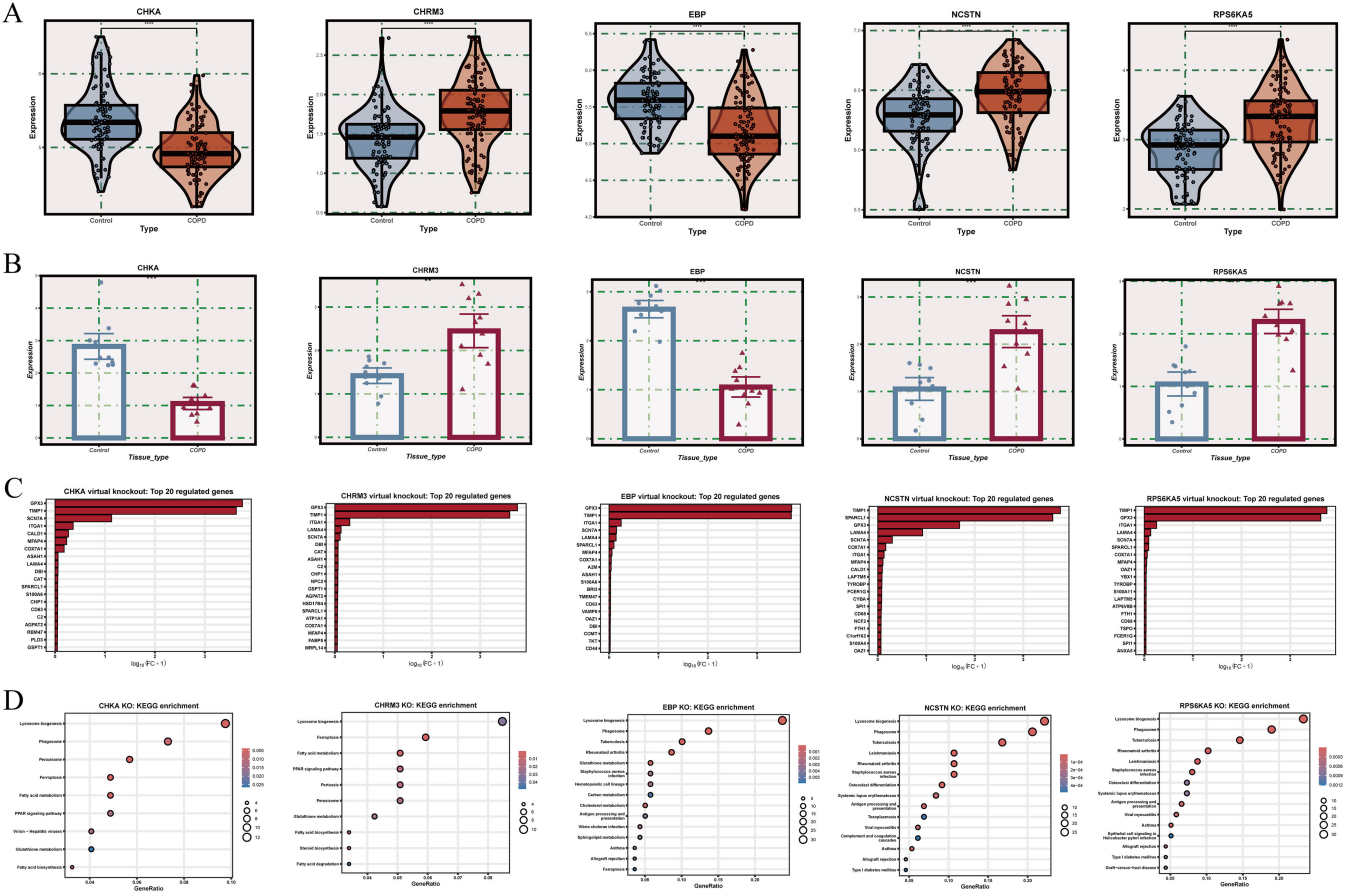
Expression validation and virtual knockout analysis of key diagnostic genes. **(A)** Expression levels of CHKA, CHRM3, EBP, NCSTN, and RPS6KA5 in the GEO COPD dataset. **(B)** RT-qPCR validation of CHKA, CHRM3, EBP, NCSTN, and RPS6KA5 expression in clinical samples. **(C)** Top regulated genes identified after virtual knockout of CHKA, CHRM3, EBP, NCSTN, and RPS6KA5 using scTenifoldKnk. **(D)** Functional enrichment analysis of genes affected by virtual knockout of each key gene.

The expression patterns of the five SVM-prioritized genes were first examined in the GEO COPD dataset. CHKA and EBP showed lower expression in COPD samples than in controls, whereas CHRM3, NCSTN, and RPS6KA5 were increased in COPD samples (**Figure 10A**). This direction was consistent with the model-derived pattern, indicating that these genes were not only selected by the diagnostic algorithm but also showed disease-associated expression shifts in the transcriptomic cohort. RT-qPCR was then performed using clinical samples to validate the expression of these genes. The PCR results reproduced the main trends observed in the GEO dataset. CHKA and EBP were reduced in COPD tissues, while CHRM3, NCSTN, and RPS6KA5 were elevated in COPD samples compared with normal controls (**Figure 10B**). These results support the expression consistency of the five genes as COPD-related diagnostic candidates. To explore the potential regulatory influence of these genes at the single-cell level, virtual knockout analysis was performed using scTenifoldKnk. After in silico knockout of CHKA, CHRM3, EBP, NCSTN, or RPS6KA5, the top dysregulated genes were ranked according to the perturbation effect. Each knockout produced a distinct set of highly affected genes, suggesting that these candidates may be associated with different regulatory networks rather than representing redundant model features (**Figure 10C**). Functional enrichment analysis of knockout-associated genes further indicated that perturbation of these key genes was linked to multiple COPD-related biological processes, including inflammatory response, immune regulation, chemokine or cytokine-related signaling, lipid and cholesterol metabolism, extracellular matrix remodeling, and receptor-mediated signaling pathways (**Figure 10D**).

As a complementary in silico perturbation analysis, scTenifoldOE was further used to perform virtual overexpression analysis for CHKA, CHRM3, EBP, NCSTN, and RPS6KA5. Virtual overexpression of different target genes generated distinct differentially regulated gene profiles, suggesting that these model-prioritized genes may not act through completely overlapping regulatory networks. After virtual overexpression of CHKA, genes such as GPX3, SPARCL1, CXCL17, SCNN1A, and MGST1 were markedly affected. After virtual overexpression of CHRM3 and NCSTN, several immune- or myeloid-related genes, including TSPO, YBX1, LAPTM5, FCER1G, CXCL16, C1QB, and TYROBP, were observed among the affected genes. Virtual overexpression of EBP and RPS6KA5 also induced gene changes related to cellular structure, adhesion, and inflammation (**Supplementary Figures 4A–E**).

GO enrichment analysis of genes affected by virtual overexpression further showed that different key gene perturbations involved distinct but partially overlapping biological functions. Genes affected by CHKA, EBP, and RPS6KA5 overexpression were mainly enriched in cell-cell junction organization, desmosome organization, cell adhesion, apical plasma membrane, basal plasma membrane, and cadherin binding, suggesting links to cell junctions, membrane structure, and adhesion-related functions. In contrast, genes affected by CHRM3 and NCSTN overexpression were more prominently enriched in antigen processing and presentation, MHC class II complex, lysosomal membrane, secretory granule, MHC protein complex binding, and peptide binding, suggesting potential involvement in antigen presentation and immune-related processes. Overall, the virtual knockout and virtual overexpression analyses provided computational evidence that these key genes may be associated with cell adhesion, immune antigen presentation, and myeloid inflammatory transcriptional programs in COPD. However, these findings remain hypothesis-generating and require further functional experimental validation.

## 4 Discussion

In this study, we integrated nicotine-related target prediction, COPD transcriptomic data, machine learning, molecular subtyping, single-cell analysis, molecular docking, virtual knockout, and RT-qPCR validation to explore nicotine-related molecular features in COPD. A nicotine-related diagnostic gene panel was identified, and the SVM model showed the best diagnostic performance. Among the model features, CHKA, EBP, RPS6KA5, NCSTN, and CHRM3 were ranked as the most important genes. These genes were further connected with immune infiltration, molecular subtype differences, myeloid-cell remodeling, and experimental validation.

Nicotine-related targets were enriched in receptor signaling, neurotransmitter-related pathways, calcium signaling, focal adhesion, ECM-receptor interaction, and PI3K-Akt signaling. These findings are consistent with the biological activity of nicotine, which can affect receptor-mediated signaling, immune regulation, epithelial responses, and tissue remodeling (31–35). After intersecting nicotine-related targets with COPD differentially expressed genes, the retained genes showed clear disease-associated expression patterns and broad correlations with immune-cell infiltration. This suggests that nicotine-related transcriptional changes may be involved in the inflammatory and immune imbalance of COPD.

The machine learning analysis identified an SVM-based model with the highest diagnostic accuracy. The top five genes have plausible biological relevance to COPD. CHKA is involved in choline metabolism and membrane lipid synthesis (36); EBP participates in sterol biosynthesis (37); RPS6KA5 is related to MAPK-dependent stress signaling (38); NCSTN is a component of the γ-secretase complex and may affect Notch-related regulation (39); and CHRM3 is closely linked to cholinergic signaling, airway contraction, and mucus secretion (40). The expression of these genes was further validated by RT-qPCR, supporting their potential value as nicotine-related diagnostic markers in COPD.

Based on SVM-derived genes, COPD samples were divided into two molecular subtypes. These subtypes showed different immune infiltration patterns and pathway activities. C1 was more associated with myeloid-cell migration, leukocyte activation, neutrophil chemotaxis, and inflammatory cytokine regulation, whereas C2 showed stronger adaptive immune and receptor-related pathway activity. This indicates that nicotine-related diagnostic genes may also reflect molecular heterogeneity within COPD.

Single-cell analysis further clarified the cellular context of the nicotine-related gene signature. The integrated gene set score was higher in epithelial cells, fibroblasts, endothelial cells, monocytes, and macrophages, suggesting that this signature is linked to both structural-cell injury and immune remodeling. Because myeloid-lineage cells showed relatively high activity, they were further reclustered. The analysis identified alveolar macrophages, monocytes, FOLR2+ macrophages, cDC2, and pDC, with functional programs related to inflammatory response, antigen presentation, lipid metabolism, and cholesterol transport. Pseudotime analysis further suggested a transcriptional continuum among myeloid-lineage cells, in which monocytes, macrophages, and alveolar macrophages occupied different regions of the inferred trajectory. However, this result should be interpreted as a state transition-like transcriptional pattern rather than direct evidence of a definitive differentiation route.

CellChat analysis showed stronger intercellular communication in the high-score group, especially involving fibroblasts, myeloid cells, endothelial cells, and epithelial cells. Pathways such as COLLAGEN, LAMININ, FN1, CCL, CXCL, TNF, MHC, and APP were more active, indicating that nicotine-related gene activity may be linked to extracellular matrix remodeling, inflammatory chemotaxis, and immune signaling. Molecular docking further provided preliminary structural clues regarding the possible binding tendency between nicotine and proteins encoded by the key model genes. However, because docking analysis is computational and several of these proteins are not classical nicotine receptors, these results should be interpreted cautiously and require further biochemical or cellular validation. In addition, virtual knockout analysis indicated that perturbation of these genes may influence inflammatory signaling, chemokine/cytokine pathways, lipid metabolism, extracellular matrix remodeling, and receptor-mediated processes.

Several limitations should be acknowledged. First, this study was mainly based on public transcriptomic datasets and a relatively limited clinical validation cohort. Although GSE57148 and GSE173896 provided valuable resources for bulk transcriptomic and single-cell analyses of COPD, the sample sizes of these datasets were still limited. This may affect the statistical power, model stability, and generalizability of the findings. Second, public datasets are inevitably affected by heterogeneity in sequencing platforms, sample processing procedures, tissue sources, disease severity, smoking exposure, and clinical background. In particular, detailed smoking-related information, such as smoking status and pack-years, was not uniformly available for all samples in the bulk transcriptomic dataset, which limited our ability to evaluate whether smoking exposure was balanced between COPD and control groups. In addition, nicotine represents only one biologically active component of tobacco exposure. Cigarette smoke contains many other toxic constituents, such as tar-associated particulate matter, aldehydes, polycyclic aromatic hydrocarbons, tobacco-specific nitrosamines, and oxidants, which may independently or synergistically contribute to COPD pathogenesis. Therefore, the nicotine-related genes identified in this study should be interpreted as a focused molecular subset associated with nicotine-related signaling, rather than as a complete representation of cigarette smoke-induced injury. Third, the single-cell dataset contained a limited number of samples, and some cell populations, especially epithelial-lineage cells, may have been underrepresented. Therefore, the distribution of nicotine-related diagnostic gene activity across different cell types should be interpreted with caution. Fourth, COPD-related DEGs and the diagnostic model were mainly derived from a single bulk transcriptomic discovery cohort. Although the key model genes were further evaluated in single-cell data and validated by RT-qPCR in clinical samples, the initial candidate gene screening and model construction may still be affected by cohort-specific factors. Further validation in larger, multicenter cohorts with harmonized preprocessing procedures and detailed clinical annotation is still required. Finally, although RT-qPCR confirmed the expression changes of CHKA, EBP, RPS6KA5, NCSTN, and CHRM3, these results do not directly demonstrate their functional roles in COPD. Future studies should include larger independent cohorts, standardized sample collection, complete smoking-exposure records, nicotine stimulation experiments, gene knockdown or overexpression, inflammatory cytokine detection, and cell functional assays to further confirm the diagnostic value and biological relevance of these nicotine-related genes. In addition, epithelial-lineage cells were relatively limited in the single-cell dataset, preventing reliable subdivision into AT1, AT2, club, ciliated, and basal epithelial subtypes. Future studies using COPD single-cell datasets with broader epithelial-cell coverage are needed to clarify nicotine-related molecular changes across epithelial subpopulations.

In summary, this study identified a nicotine-related diagnostic gene panel for COPD and linked it to immune infiltration, molecular subtypes, cell-cell communication, and myeloid-cell remodeling. These findings should be interpreted as nicotine-focused transcriptomic associations within the broader context of tobacco smoke-related COPD biology.

## Data Availability

The original contributions presented in the study are included in the article/**Supplementary Material**. Further inquiries can be directed to the corresponding author.

## Supplementary material

Supplementary Figure 1 Correlation analysis of the five core diagnostic genes. Spearman correlation heatmap showing pairwise expression correlations among CHKA, EBP, NCSTN, RPS6KA5, and CHRM3 across all included samples. Numbers indicate Spearman correlation coefficients, and asterisks indicate statistical significance.

Supplementary Figure 2 Cell–cell communication patterns between NRGs-low and NRGs-high groups. **(A)** Heatmaps showing outgoing signaling patterns across major cell types in the NRGs-low and NRGs-high groups. **(B)** Heatmaps showing incoming signaling patterns across major cell types in the NRGs-low and NRGs-high groups. **(C, D)** Scatter plots showing the outgoing and incoming interaction strength of each cell type in the NRGs-low and NRGs-high groups.

Supplementary Figure 3 Classical marker expression of myeloid subpopulations. Dot plot showing representative marker expression for alveolar macrophages, monocytes, FOLR2+ macrophages, cDC2, and pDC in the reclustered myeloid compartment. Dot color indicates scaled average expression, and dot size indicates the percentage of marker-expressing cells.

Supplementary Figure 4 Transcriptional perturbation and functional enrichment analysis after virtual overexpression of key genes. **(A–E)** Results of virtual overexpression analysis for CHKA, CHRM3, EBP, NCSTN, and RPS6KA5, respectively. For each panel, the upper plot shows the top 20 differentially regulated genes after virtual overexpression, and the lower plot shows GO enrichment analysis of the corresponding affected genes. The x-axis in the bar plots represents log10(FC + 1), and the x-axis in the enrichment plots represents −log10(adjusted P value). GO enrichment terms are displayed according to BP, CC, and MF categories.

Supplementary Table 1 RT-qPCR validation samples clinical characteristics.

Supplementary Table 2 primer sequences.

Supplementary Table 3 Nicotine-related target records retrieved from the ChEMBL database.

Supplementary Table 4 Nicotine-related target genes predicted by SwissTargetPrediction.
